# Evaluating the quality and use of economic data in decisions about essential medicines

**DOI:** 10.2471/BLT.14.149914

**Published:** 2015-08-14

**Authors:** Corrina Moucheraud, Veronika J Wirtz, Michael R Reich

**Affiliations:** a Harvard TH Chan School of Public Health, Boston, Department of Global Health and Population, 665 Huntington Avenue, Building 1, Boston, Massachusetts 02115, United States of America (USA).; b Boston University School of Public Health, Department of Global Health, Boston, USA.

## Abstract

**Objective:**

To evaluate the quality of economic data provided in applications to the World Health Organization (WHO) Model List of Essential Medicines and to evaluate the role of these data in decision-making by the expert committee that considers the applications.

**Methods:**

We analysed applications submitted to the WHO Expert Committee on the Selection and Use of Essential Medicines between 2002 and 2013. The completeness of data on the price and cost–effectiveness of medicines was extracted from application documents and coded using a four-point scale. We recorded whether or not the expert committee discussed economic information and the outcomes of each application. Associations between the completeness of economic data and application outcomes were assessed using *χ^2^* tests.

**Findings:**

The expert committee received 134 applications. Only eight applications (6%) included complete price data and economic evaluation data. Many applicants omitted or misinterpreted the economic evaluation section of the application form. Despite the lack of economic data, all applications were reviewed by the committee. There was no significant association between the completeness of economic information and application outcomes. The expert committee tried to address information gaps in applications by further review and analysis of data related to the application.

**Conclusion:**

The World Health Organization should revise the instructions to applicants on economic data requirements; develop new mechanisms to assist applicants in completing the application process; and define methods for the use of economic data in decision-making.

## Résumé

**Objectif:**

Évaluer la qualité des données économiques fournies dans les candidatures à la Liste Modèle des Médicaments Essentiels de l'Organisation Mondiale de la Santé (OMS) et évaluer le rôle de ces données dans la prise de décision du comité d'experts chargé d'examiner les candidatures.

**Méthodes:**

Nous avons analysé les candidatures soumises au comité d'experts de l'OMS concernant la sélection et l'utilisation des médicaments essentiels entre 2002 et 2013. Toutes les données relatives aux prix et au rapport coût-efficacité des médicaments ont été tirées des dossiers de candidature et leur exhaustivité a été codée à l'aide d'une échelle à quatre points. Nous avons déterminé si le comité d'experts examinait les informations économiques et l'issue de chaque candidature. La relation entre le caractère exhaustif des données économiques et l'issue des candidatures a été évaluée par des tests du *χ^2^*.

**Résultats:**

Le comité d'experts a reçu 134 candidatures. Seules huit d'entre elles (6%) incluaient des données complètes sur les prix et des données d'évaluation économique. De nombreux candidats ont négligé ou mal compris la section du formulaire de candidature relative à l'évaluation économique. Malgré le manque de données économiques, toutes les candidatures ont été examinées par le comité. Aucune relation significative entre l'exhaustivité des informations économiques et l'issue des candidatures n'a été établie. Le comité d'experts a tenté de remédier à l'insuffisance des informations fournies dans les candidatures en procédant à un examen et à une analyse plus poussés des données relatives à la candidature.

**Conclusion:**

L’Organisation Mondiale de la Santé doit revoir les indications données aux candidats en ce qui concerne les exigences relatives aux données économiques, élaborer de nouveaux mécanismes pour aider les candidats à aller au bout du processus de candidature et définir des méthodes d'utilisation des données économiques dans la prise de décision.

## Resumen

**Objetivo:**

Evaluar la calidad de los datos económicos proporcionados en las solicitudes para la Lista Modelo de Medicamentos Esenciales de la Organización Mundial de la Salud (OMS) y valorar el papel que desempeña dicha información en el proceso de toma de decisiones del Comité de Expertos que tiene en cuenta las solicitudes.

**Métodos:**

Se analizaron las solicitudes entregadas al Comité de Expertos de la OMS en relación con la selección y el uso de medicamentos esenciales entre 2002 y 2013. La exactitud de los datos sobre el precio y la costoeficacia de los medicamentos se extrajo de los documentos de solicitud y se calificó mediante una escala de cuatro puntos. Se registró si en el Comité de Expertos se discutió o no la información económica y los resultados de cada solicitud. Las asociaciones entre la exactitud de los datos económicos y los resultados de las solicitudes se evaluaron usando pruebas *χ^2^*.

**Resultados:**

El comité de expertos recibió 134 solicitudes. Solamente ocho solicitudes (6%) incluían datos completos relativos al precio y datos relativos a la evaluación económica. Muchos solicitantes omitieron o malinterpretaron la sección de evaluación económica del formulario de solicitud. A pesar de la falta de datos económicos, el Comité revisó todas las solicitudes. No hubo ningún vínculo significativo entre la exactitud de la información económica y los resultados de la solicitud. El Comité de Expertos trató de remediar la falta de información en las solicitudes mediante una revisión más exhaustiva y un análisis de los datos relacionados con dicha solicitud.

**Conclusión:**

La Organización Mundial de la Salud deberá revisar las instrucciones para los solicitantes que figuran en los requisitos de los datos económicos, desarrollar nuevas formas de asistir a los solicitantes a la hora de completar el proceso de solicitud y definir métodos para el uso de datos económicos en el proceso de toma de decisiones.

## ملخص

الغرض تقييم مستوى جودة البيانات الاقتصادية المقدمة في طلبات الإدراج ضمن القائمة النموذجية للأدوية الأساسية بمنظمة الصحة العالمية (WHO)، وتقييم الدور الذي تلعبه هذه البيانات في اتخاذ القرارات من جانب لجنة الخبراء التي تنظر في الطلبات.

الطريقة لقد قمنا بتحليل الطلبات المقدمة للجنة الخبراء بمنظمة الصحة العالمية بشأن اختيار الأدوية الأساسية واستخدامها في الفترة ما بين عامي 2002 و2013. وتم استخلاص درجة اكتمال البيانات المتعلقة بسعر الأدوية ومعقولية تكلفتها من خلال المستندات التي تضمنتها الطلبات، كما تم التعبير عن ذلك بالترميز، مع الاستعانة بمعيار من أربع نقاط. وسجلنا ما إذا كانت لجنة الخبراء قد قامت بمناقشة المعلومات الاقتصادية والنتائج فيما يتعلق بكل طلب، أم لا. وتم تقييم حالات الارتباط بين درجة اكتمال البيانات الاقتصادية من جهة، ونتائج الطلبات من جهة أخرى، وذلك باستخدام اختبارات خي مربع *χ ^2 ^*.

النتائج تسلمت لجنة الخبراء 134 طلباً. وتضمنت ثمانية طلبات فقط (6%) بيانات مكتملة عن السعر وبيانات عن التقييم الاقتصادي. وقد حذف الكثير من مقدمي الطلبات قسم التقييم الاقتصادي بنموذج الطلب أو أساءوا تفسيره. وبرغم نقص البيانات الاقتصادية، قامت اللجنة بمراجعة جميع الطلبات. ولم يوجد أي ارتباط واضح بين درجة اكتمال المعلومات الاقتصادية، ونتائج الطلب، بل حاولت لجنة الخبراء سد فجوات البيانات في الطلبات المقدمة عن طريق إجراء المزيد من المراجعة والتحليل للبيانات المتعلقة بالطلب.

الاستنتاج يجب على منظمة الصحة العالمية مراجعة التعليمات الخاصة بمقدمي الطلبات، والمتعلقة بمتطلبات البيانات الاقتصادية، كما يجب عليها وضع آليات جديدة لمساعدة مقدمي الطلبات في إكمال إجراءات تقديم الطلب، وكذلك تحديد بعض الطرق لاستخدام البيانات الاقتصادية في اتخاذ القرارات.

## 摘要

目的 评估向世界卫生组织 (WHO) 申请的基本药物标准清单中所提供的经济数据的特性。同时也评估专家委员会审议申请时，这些数据在他们制定决策中所起的作用。

方法 我们分析了在 2002 年至 2013 年间提交给 WHO 专家委员会的关于选择和使用基本药物的申请。药物价格和成本效益的数据完整性从申请文件中提取，并且用四点量表编码。 我们记录了专家委员会是否讨论了经济信息以及每份申请的结果。 我们用 *χ^2^* 检验对经济数据的完整性和申请结果之间的关联性进行了评估。

结果 专家委员会共收到 134 份申请。 只有8 份申请 (6%) 包含完整的价格数据和经济评估数据。 许多申请都遗漏或曲解了申请表的经济评估部分。 尽管缺少经济数据，但委员会还是审查了所有的申请。 经济信息的完整性和申请结果之间并无重大关联。 专家委员会尽力通过进一步审查和分析申请相关数据来处理申请中的信息缺失。

结论 世界卫生组织应该修改供申请人查阅的经济数据要求说明，制定有助于申请人完成申请过程的新机制，规定决策中经济数据起作用的方法。

## Резюме

**Цель:**

Оценить качество экономических данных, предоставленных в заявках на примерный список основных лекарств, составленный Всемирной организацией здравоохранения (ВОЗ), и оценить роль этих данных при принятии комитетом экспертов решений по этим заявкам.

**Методы:**

Мы проанализировали заявки, подаваемые в комитет экспертов ВОЗ по отбору и использованию основных лекарств за период между 2002 и 2013 гг. Полный объем данных о ценах и соотношении эффективности и расходов для конкретных лекарств были получены по документации, прилагаемой к заявкам. Данный объем оценивается по четырехбалльной шкале. Мы также отметили наличие или отсутствие обсуждения экономической информации комитетом экспертов и то, какое решение было принято по каждой из заявок. Связь между полнотой экономических данных и результатом рассмотрения заявки была оценена по критерию *χ^2^*.

**Результаты:**

Комитет экспертов получил за это время 134 заявки. Из них только в восьми (6%) имелись полные данные о ценах и экономическая оценка. Во многих заявках раздел экономической оценки был не заполнен или заполнен неправильно. Несмотря на недостаток экономической информации, комитет рассмотрел каждую из заявок. Связи между полнотой экономической информации и результатом рассмотрения заявки выявлено не было. Комитет экспертов пытался заполнить пробелы в заявках путем дальнейшего изучения и анализа данных, связанных с отдельной заявкой.

**Вывод:**

Комитету экспертов следует пересмотреть инструкции, выдаваемые заявителям, в части требований, касающихся экономических данных, разработать новые механизмы помощи заявителям в заполнении заявок, а также определить методы использования экономических данных при принятии решений.

## Introduction

Essential medicines are those that satisfy the priority health care needs of the population.^1^ The World Health Organization (WHO) introduced the first Model List of Essential Medicines in 1977. Countries are encouraged to use the model list as a guide for their decisions on pharmaceutical selection and procurement. Between 1977 and 2007, over 130 countries introduced national lists of essential medicines, modifying the WHO model list for their national context.^2^

The model list is updated every two years, following an application and review process by an expert committee. Any individual or institution may submit an application. Before each committee meeting, applications are published on a website for public comment and experts (committee members and external advisors) review each application.^3^ These reviews and any public comments are also published on the website before the committee meeting. Since 2002, essential medicines have been selected via an evidence-based process, with due regard to public health relevance, evidence on efficacy and safety and comparative cost–effectiveness.^1^ Detailed information on the decision criteria can be found at http://www.who.int/selection_medicines/en/. The model list is divided into a core list and a complementary list. The core list includes medicines that meet the criteria of efficacy, safety and comparative cost–effectiveness,^1^ but expensive patented medicines are not necessarily excluded.^3^^,^^4^ The complementary list includes medicines that may require specialized facilities, that are consistently more costly, or less cost–effective in a variety of settings.^5^

Additions to the model list can have a major impact on global and national decisions, with significant budgetary, ethical and health implications.^6^^,^^7^ Other studies have examined application processes and criteria for national decisions on adopting new vaccines,^8^ national health technology assessment programmes^9^^,^^10^ and financing decisions for health technologies.^11^ A recent study found that applications to the model list for mental health medicines generally provided low-quality and incomplete evidence across several required dimensions.^12^ There is no global consensus on how to use economic data in decision-making for medicines and health technologies.^13^^–^^17^

Here, we evaluate the extent to which applicants and the expert committee adhere to the instructions and procedures on economic data and analysis for applications to the model list. We assess the application process rather than the substance of decisions (specifically, compliance with instructions for applicants and the quality of data on price and on cost–effectiveness). We also assess whether economic considerations are included in the final report by the expert committee. The overall goal of this study is to improve the quality, transparency and clarity of the process for reviewing applications to the model list.

## Methods

### Data set

We included applications to the model list for medicines intended for use in adults. We analysed final reports from the twelfth (2002) to nineteenth (2013) meetings of the WHO expert committee; the sixteenth meeting reviewed paediatric medicines only and was not included. We did not review applications for new formulations of existing medicines or applications for reinstatement.

### Definition of variables

We assessed the extent to which applicants complied with the instructions provided. The instructions ask applicants to provide “a range of costs of the proposed medicine” that reflect “average generic world market prices”, with a clearly specified source of data. The WHO-recommended sources for these data include the International Drug Price Indicator Guide, the United Nations Children’s Fund, Médecins Sans Frontières or WHO itself. We allocated one point for each of the following: price given; offered range of price or average or median price; clear source; WHO-recommended source. The application was classified as complete if the application attained a score of four; applications with scores between one and three points (inclusive) were classified as incomplete. If no pricing data were provided, this was considered missing (zero points). If the applicant explicitly mentioned that no pricing data exist, this was classified as not available.

Completeness of economic evaluation data was scored based on WHO instructions for comparative cost–effectiveness presented as range of cost per routine outcome, and a clear citation, preferably of WHO-recommended sources. We also accepted citations from the published scientific literature as one of the recommended data sources. Adherence was classified as complete if applications met all of the following criteria: economic evaluation value provided; comparator given; clear source; WHO-recommended source. As with price, applications scoring between one and three inclusive were classified as incomplete. Omitted economic evaluation sections were classified as missing; cases where the applicant explicitly noted the lack of available data on economic evaluation were classified as not available. There were instances in which the applicant provided administration costs for medicines, without an outcomes-based denominator, under the heading of cost–effectiveness. These applications were classified as having provided economic evaluation data that were not applicable.

Based on the final reports from each meeting, the outcome was coded as a dichotomous variable: either rejected or deferred (coded 0) versus accepted to the core or complementary lists (coded 1). Applicant type and therapeutic class were categorized based on information provided in the application. We recorded the proportion of applications reviewed by the committee that were added to the model list, by therapeutic class. We also recorded whether the discussion and decisions mentioned price or economic evaluation.

### Data entry and analysis

A data entry form was generated based on the variables listed above. There were two independent data reviewers, each of whom extracted data from the applications; discrepancies were resolved by consensus. Application data were entered using CSPro version 5 (United States Census Bureau, Washington, United States of America).

Qualitative information from discussions and application decision categories were coded by hand. We extracted the narrative sections about the reviewed medicines from the final report for each committee meeting; text concerning economic considerations was collated in an Excel spreadsheet. We identified whether the report mentioned economic data or the absence of such data, as well as which data sources were cited (i.e. the application itself or new additional data).

Associations between the completeness of economic data and the application outcomes were assessed using *χ^2^* tests in Stata version 12.1 (StataCorp. LP, College Station, USA). We conducted sensitivity analyses that dropped each scoring criterion in turn.

## Results

We analysed 134 applications for new medicines from 2002 to 2013 (Table 1). Application authors varied by year, but were most commonly submitted by WHO departments (25.4%; 34/134), academic institutions (22.4%; 30/134) and nongovernmental organizations (21.6%; 29/134). Therapeutic classes of medicines also varied between meetings; applications for cancer and diabetes medicines were more common in recent years. Of 134 applications analysed, 52 were rejected and decisions were deferred for an additional 10. Among the 72 applications added to the model list, 67 were added to the core list and five to the complementary list.

**Table 1:** Applications to the WHO Expert Committee on the Selection and Use of Essential Medicines, 2002–2013

Application characteristic	No. of applications (%)							
	Meeting of the WHO Expert committee (year)							Total
	12th (2002)	13th (2003)	14th (2005)	15th (2007)	17th (2009)	18th (2011)	19th (2013)	
Applications	16 (100)	7 (100)	17 (100)	22 (100)	35 (100)	17 (100)	20 (100)	134 (100)
Additions to the list	11 (68.8)	3 (42.9)	7 (41.2)	19 (86.4)	12 (34.3)	10 (58.8)	10 (50.0)	72 (53.7)
**Applicant**								
WHO (internal)	12 (75.0)	4 (57.1)	2 (11.8)	7 (31.8)	4 (11.4)	3 (17.6)	2 (10.0)	34 (25.4)
Academia	0 (0.0)	1 (14.3)	1 (5.9)	3 (13.6)	15 (42.9)	6 (35.3)	4 (20.0)	30 (22.4)
NGO	0 (0.0)	2 (28.6)	5 (29.4)	3 (13.6)	11 (31.4)	0 (0.0)	8 (40.0)	29 (21.6)
Industry	3 (18.8)	0 (0.0)	5 (29.4)	6 (27.3)	0 (0.0)	6 (35.3)	1 (5.0)	21 (15.7)
Other^a^	1 (6.2)	0 (0.0)	4 (23.5)	3 (13.6)	5 (14.3)	2 (11.8)	5 (25.0)	20 (14.9)
**Class of medicine**								
HIV	10 (62.5)	0 (0.0)	3 (17.6)	7 (31.8)	2 (5.7)	3 (17.6)	0 (0.0)	25 (18.7)
Infections	1 (6.3)	4 (57.1)	3 (17.6)	4 (18.2)	5 (14.3)	2 (11.8)	2 (10.0)	21 (15.7)
Mental health	0 (0.0)	0 (0.0)	1 (5.9)	1 (4.5)	14 (40.0)	0 (0.0)	2 (10.0)	18 (13.4)
Tuberculosis and malaria	4 (25.0)	1 (14.3)	0 (0.0)	2 (9.1)	1 (2.9)	3 (17.6)	2 (10.0)	13 (9.7)
Cancer	0 (0.0)	0 (0.0)	0 (0.0)	1 (4.5)	4 (11.4)	3 (17.6)	4 (20.0)	12 (9.0)
RH and MCH	0 (0.0)	1 (14.3)	7 (41.2)	2 (9.1)	2 (5.7)	0 (0.0)	0 (0.0)	12 (9.0)
CVD and diabetes	0 (0.0)	1 (14.3)	0 (0.0)	1 (4.5)	1 (2.9)	2 (11.8)	1 (5.0)	6 (4.5)
Other^b^	1 (6.3)	0 (0.0)	3 (17.6)	4 (18.2)	6 (17.1)	4 (23.5)	9 (45.0)	27 (20.2)

CVD: cardiovascular disease; HIV: human immunodeficiency virus; MCH: maternal and child health; NGO: nongovernmental organization; RH: reproductive health; WHO: World Health Organization.

^a^ Government groups and co-applicants of different types (e.g. NGO-academic partnerships).

^b^ Includes analgesics, antidotes, gastrointestinal medicines, antivirals, anaesthesia and sedatives, ophthalmology preparations and nutritional supplements including vitamins and minerals.

Across all meeting years, only 6.0% of applications (8/134) provided complete price and economic evaluation data (Table 2). Since no time trends were apparent, we report totals for all years; analyses by year are available from the corresponding author. If we include applications that reported economic data were not available, assuming that these applicants made an effort to include data but that none were available, 13.4% (18/134) of applications fully followed the instructions on price and economic evaluation. Only 20.9% (28/134) and 18.7% (25/134) of applications provided complete price and economic evaluation information, respectively. Over a third (36.6%; 49/134) included no economic evaluation information, one-fifth (20.9%; 28/139) stated that economic evaluation information was unavailable and 17.9% (24/134) presented financial information that was unrelated to economic evaluation (e.g. total treatment cost).

**Table 2:** Completeness of economic information provided in applications to the WHO Expert Committee on the Selection and Use of Essential Medicines, 2002–2013

Application status	No. of applications (%)^a^									
	Price (*n* = 134)					Economic evaluation (*n* = 134)				
	Complete	Incomplete	Not available	Missing		Complete	Incomplete	Not available	Not applicable	Missing
**Applied**	28 (20.9)	79 (59.0)	7 (5.2)	20 (14.9)		25 (18.7)	8 (6.0)	28 (20.9)	24 (17.9)	49 (36.6)
**Added to list**	18 (64.3)	43 (54.4)	4 (57.1)	7 (35.0)		13 (52.0)	5 (62.5)	14 (50.0)	12 (50.0)	28 (57.1)

WHO: World Health Organization.

^a^ If applicant explicitly noted that price and/or economic evaluation data did not exist for the medicine, these were classified as not available. To be classified as missing, the applicant did not provide any price or economic evaluation information. Applications classified as not applicable were those in which the economic evaluation sections did not include outcomes-based measures (i.e. neither cost–effectiveness, cost–benefit nor cost–utility analysis).

All applications were reviewed by the committee. For the applications with complete economic data, 52.0% (13/25) was added to the model list. When we included applications that stated that economic data were not available, 50.9% (27/53) was added to the model list. Among applications with no price data or no economic evaluation, over one-third and over half, respectively, of these applications were added to the model list.

There was variation in the extent of economic information provided by applicant type. Academic authors were most likely to provide complete price and economic evaluation information. Applicants from WHO did not include economic evaluation information in half of their applications and industry applicants very rarely provided complete information on price or economic evaluation. Detailed data on the completeness of economic information by applicant type and therapeutic class are available from the corresponding author.

For applications with complete price data, 64.3% (18/28) were added to the model list, a percentage that was not significantly higher than for applications with incomplete or absent price information (50.5%; 50/99; *P* = 0.2). For applications with complete economic evaluation information 52.0%, (13/25) were added to the model list, compared to 54.1% for those with incomplete, missing, inapplicable or unavailable information (59/109; *P* = 0.9).

We explored whether the committee’s discussions of economic factors reflected the content of the applications. As shown in Table 3, price data or economic evaluations were discussed in some cases, even where this information had not been included in the application. Our qualitative analysis suggests four ways in which this happened. First, the committee sometimes noted the lack of information in the application (two applications with missing price data, seven applications with missing economic evaluation); in three of these cases, the medicines were nonetheless added to the core list. Second, the committee sometimes referenced economic information, suggesting an alternative, although unspecified, source of supplementary data (six applications). Third, the committee sometimes did further research: for example, the report mentioned a review of cost–effectiveness data prepared by the secretariat, or indicated that the secretariat (usually comprised of WHO staff who support the committee process) performed its own economic evaluation (four applications). Fourth, in two instances where an economic evaluation was missing, the committee discussed price data included in the application but referred to it as “cost–effectiveness,” suggesting a misinterpretation.

**Table 3:** Essential medicine list applications where price or economic evaluation are mentioned in final reports from the WHO Expert Committee on the Selection and Use of Essential Medicines, 2002–2013

Type	No. of applications (%)			
	Complete information	Incomplete information	No information^a^	Total
**Price**				
Not mentioned	14 (50)	44 (56)	20 (74)	78 (58)
Mentioned	14 (50)	35 (44)	7 (26)	56 (42)
Total	28 (100)	79 (100)	27 (100)	134 (100)
**Economic evaluation**				
Not mentioned	10 (40)	4 (50)	79 (78)	93 (69)
Mentioned	15 (60)	4 (50)	22 (22)	41 (31)
Total	25 (100)	8 (100)	101 (100)	134 (100)

WHO: World Health Organization.

^a^ Applications where no information was provided for price and economic evaluation, plus those where the applicant explicitly noted that price and economic evaluation data did not exist for the medicine and those earlier classified as not applicable.

We explored whether the distinction between core and complementary lists was followed in the decision process. A feature that distinguishes the complementary list is its inclusion of medicines requiring specialized diagnostic or monitoring facilities and/or specialist medical care and/or specialist training.^5^ According to our analysis, 50% (46/92) of applications that we classified as belonging to the complementary list were nonetheless added to the core list by the committee. This included all psychiatric medicines and antiretroviral medicines for treating human immunodeficiency virus infections.

We conducted sensitivity analyses, to examine the relative importance of each scoring criterion. The most influential variables were: for price data, the average generic world price and for economic evaluations, the choice of comparator (i.e. presenting relative or incremental data). The fourth criterion, use of a WHO-recommended source for economic data, might be seen as an optional point: dropping this criterion from the analysis did not substantially increase the number of applications scored as complete (from 20.9% complete on price to 28.4% and from 18.7% complete for economic evaluation to 19.4%).

## Discussion

Very few applications complied fully with the instructions on providing price data and economic evaluations. The quality of information provided in applications to the Model List of Essential Medicines can be improved substantially. Despite the majority of applications being submitted without the required economic information, the committee reviewed all applications. There was no significant association between the completeness of economic information and the outcome of applications: provision of economic data in an application is not necessary for a positive decision by the committee.

Our qualitative findings suggest that in some cases, the committee found alternative ways to address information gaps – such as internal literature reviews and analyses. However, such data collection and analysis requires additional time on the part of the committee and reviewers. Since the committee still considered and approved incomplete applications, this may have reinforced applicants’ decisions to not comply with instructions. There is little evidence that applicants have improved their efforts to submit requested information over time.

There are several possible reasons why applicants often did not provide complete or robust economic data. First, this information may be hard to collect: high-quality price and economic evaluation data that are relevant for resource-poor settings may be difficult to find in the literature; applicants may lack the technical skills to conduct their own economic evaluation. Such issues should, however, be less of a barrier for applicants from private companies, who have access to proprietary data, but applications from companies had some of the lowest rates of provision of economic information. Second, the application instructions may be unclear or insufficiently detailed, as suggested at a recent committee meeting. Application instructions for the recent twentieth committee meeting were revised,^18^ but still do not fully incorporate recent recommendations, such as asking applicants to provide comprehensive search strategies or stating that only complete applications would be reviewed. Third, the committee itself may not be clear about whether economic studies should be a necessary component of the decision process. Our qualitative analysis suggests that other considerations – such as safety and tolerability – are sometimes given higher priority even in cases of unfavourable price or economic evaluation data. However, it is often unclear which criteria were emphasized during the committee’s review and decision process. There are also challenges in using economic criteria, such as comparative cost–effectiveness, in decision-making for medicines. These challenges include information deficiencies and a lack of universal standards on appropriate thresholds.

Our study has some limitations. We did not assess the accuracy of information provided and when an applicant noted that no data were available, we did not attempt to verify this. Our analysis of committee discussions and decisions was limited to the publicly-available meeting reports, which may not capture all aspects of the meeting. To overcome the various data limitations, we relaxed certain criteria in the application instructions to allow more flexibility in assessing adherence to the instructions: for example, we expanded the average generic world market price criterion to permit inclusion of median prices and prices per country-group (e.g. low-income countries); we allowed other published data sources (e.g. peer-reviewed manuscripts) or internal sources (e.g. clinical trial dossiers) for economic evaluation.

In conclusion, we have three recommendations to improve the review and decision-making process for the Model List of Essential Medicines. WHO should: (i) provide clear and detailed instructions about how much and what kind of economic data are required; (ii) develop mechanisms to assist applicants in completing an application; and (iii) provide clear rules about how economic data will be used in making decisions about applications and about the consequences of not providing economic data (Box 1). To implement these recommendations, the current application form needs to be revised and examples of high-quality applications and an interactive, guided, application process should be provided. Many countries are debating how to use economic data and analysis in decision-making processes about essential medicines. WHO could assist these countries by providing clear examples of how applications should be prepared and how decisions should be reached. The model list is an important global tool and decisions about new additions have significant implications for national policies and budgets, as well as clinical decision-making.

Box 1 **Detailed recommendations for the WHO Expert Committee on the Selection and Use of Essential Medicines**

1. The World Health Organization (WHO) should provide clear and detailed application instructions on how much and what kind of economic data (price data and economic evaluation information) should be presented, based on accepted standards.^19^
2. For price data, the current instructions ask applicants to provide a “range of costs of the proposed medicine and to show medicine prices from a range of settings where the product is registered.” This should be clarified: a range of unit prices (not costs) should be provided for specific countries and for specific sellers and data should include low- and/or middle-income countries whenever possible. The application should clearly identify the source of price data, the years for the data and conditions that apply to the prices (bulk purchasing, payment method, etc.). In addition to unit prices, the applicant should provide meaningful per-patient prices, for example, per treatment duration or per full vaccination course. If no price data exist, this should be explicitly noted as such and the efforts to find price data should be documented.
3. For economic evaluations (cost–effectiveness, cost–benefit and cost–utility analyses), applications should provide clear units (for both costs and outcomes), all comparisons (ideally reported as incremental costs and outcomes) and citations and the relevant country or countries. The economic evaluation should include information on administration requirements (human resources, supplies) and costs for these if possible. If any data components are lacking in the literature, this should be explicitly noted. For published economic evaluations, applicants should provide details of their search strategy (sources and keywords used). All results of this search should be presented in full. For applications reporting new economic evaluation analyses, applicants should include a full accounting of all cost and outcome data components and sources, plus information on the modelling approach and sensitivity and uncertainty analyses. Applicants should provide a discussion and interpretation of the results presented.
4. For both price data and economic evaluation information, the distinction between required and optional information should be explicitly stated in the application instructions. For price data, required items might include unit and clinical prices (e.g. per course of treatment); and for economic evaluation, clear outcomes and comparative, incremental analyses (e.g. incremental cost–effectiveness ratios).
5. For price data reported directly in the application and for any price data used in de-novo economic evaluation calculations, WHO should seek to extend its partnerships with agencies that collect economic information – currently WHO recommends data sources such as the International Drug Price Indicator Guide and the United Nations Children’s Fund. This is a longer-term goal, but would provide a standardized, high-quality source for applications that present price data.
6. WHO should develop mechanisms to assist prospective applicants in completing an application. We recommend four new mechanisms, which may also require additional financing.
7. The application instructions should be reviewed using focus groups and feedback from past applicants and reviewers, to improve the clarity of instructions around data types and formats (as suggested in recommendation 1a–d).
8. Examples of high-quality, complete applications from the past should be provided to prospective applicants, to illustrate appropriate responses to specific sections of the application instructions.
9. An interactive, guided, form-based application process, with detailed application sections and mandatory fields, could be developed to improve the process of applying and assure that all sections of an application are completed before submission.
10. WHO should develop an online training course and require that this course be completed by applicants before submission of a new application. This would include information about the model list, its objectives and role, as well as technical guidance in completing a high-quality application, including identifying, interpreting and using economic data. The World Health Assembly passed a resolution to improve policies and practices for adopting national-level model lists, including promoting collaboration and information-sharing about best practices for selection procedures^20^; WHO could extend such technical assistance and education programmes to aid national decision processes as well.
11. WHO should provide clear rules about how economic data will be used in making decisions and about the consequences of not providing economic data.
12. Incomplete applications should be identified as such and should not be reviewed. Applicants should be informed that their application is not complete and will not be reviewed unless all instructions are met, including providing specific price data and economic evaluation studies as identified via recommendation 1c.
13. All criteria used to guide application assessments should be accounted for in documentation of the committee’s decisions: just as a structured application form/process could help ensure uniform provision of information, a template of the process for committee decisions could increase transparency. For instance, the committee report should provide a brief snapshot summary of information provided in terms of the medicine’s safety, efficacy, quality and economic profile. A model for this already exists, in the expert review process before the committee meeting – and this type of form could be expanded and incorporated into the committee decision process. There is a short timeline after each meeting in which the report is generated (with decision justifications, etc.); standardized reporting might facilitate a quicker and more efficient process for writing the report, but rapid turnaround should be considered as an important factor in adopting such a change.
14. WHO should provide clear decision rules about what information and criteria are used to assign an application for consideration under the core list or the complementary list, including the role of economic data in doing so. These determinations should also be clearly accounted for in documentation of the committee’s decisions.
15. All documents used in the application process before the committee meeting should be publicly available on a WHO website, including initial application, all expert reviews, any application revisions, all public comments and all additional information sought from applicants or others. All information used in the process should be fully documented and publicly available, including details of the committee’s decision process, including assessment of information provided as per items 3a–b and any additional data or analyses prepared by the Secretariat, to assure full transparency about how decisions are made and to strengthen the evidence base. The committee should continue to require only publicly-available information in applications.
